# Eye Movement Classification Using Neuromorphic Vision Sensors

**DOI:** 10.3390/jemr19010017

**Published:** 2026-02-04

**Authors:** Khadija Iddrisu, Waseem Shariff, Maciej Stec, Noel O’Connor, Suzanne Little

**Affiliations:** 1 Faculty of Engineering and Computing, Dublin City University, D09DXA0 Dublin, Ireland; 2 C3I Imaging Lab, School of Engineering, University of Galway, H91TK33 Galway, Ireland; waseem.shariff@universityofgalway.ie (W.S.);

**Keywords:** eye movements, event cameras, spiking neural networks

## Abstract

Eye movement classification, particularly the identification of fixations and saccades, plays a vital role in advancing our understanding of neurological functions and cognitive processing. Conventional modalities of data, such as RGB webcams, often face limitations such as motion blur, latency and susceptibility to noise. Neuromorphic Vision Sensors, also known as event cameras (ECs), capture pixel-level changes asynchronously and at a high temporal resolution, making them well suited for detecting the swift transitions inherent to eye movements. However, the resulting data are sparse, which makes them less well suited for use with conventional algorithms. Spiking Neural Networks (SNNs) are gaining attention due to their discrete spatio-temporal spike mechanism ideally suited for sparse data. These networks offer a biologically inspired computational paradigm capable of modeling the temporal dynamics captured by event cameras. This study validates the use of Spiking Neural Networks (SNNs) with event cameras for efficient eye movement classification. We manually annotated the EV-Eye dataset, the largest publicly available event-based eye-tracking benchmark, into sequences of saccades and fixations, and we propose a convolutional SNN architecture operating directly on spike streams. Our model achieves an accuracy of 94% and a precision of 0.92 across annotated data from 10 users. As the first work to apply SNNs to eye movement classification using event data, we benchmark our approach against spiking baselines such as SpikingVGG and SpikingDenseNet, and additionally provide a detailed computational complexity comparison between SNN and ANN counterparts. Our results highlight the efficiency and robustness of SNNs for event-based vision tasks, with over one order of magnitude improvement in computational efficiency, with implications for fast and low-power neurocognitive diagnostic systems.

## 1. Introduction

Eye movements, particularly fixations and saccades, are fundamental components of visual perception, and they provide valuable insights into cognitive function and neurological health. Their classification is critical in diverse domains, ranging from neuroscience and psychology to human–computer interaction and assistive technologies. Traditional modalities for tracking and analyzing eye movements, such as scleral search coils [1], video-based eye trackers [2], and electroencephalogram (EEG) [3], while effective, are often constrained by limitations including motion blur, latency, low dynamic range, and susceptibility to environmental conditions.

Different classes of eye movements, such as saccades, smooth pursuit, and fixations, can be categorized by their roles in vision, physiological properties, and neural mechanisms. These distinctions enable researchers to link specific eye movement patterns to particular neurological functions and dysfunctions [4]. Their importance extends beyond vision; they provide a window into cognitive processes, personality traits, and even the progression of neurological diseases. Advances in eye-tracking technologies have further enhanced their utility as objective biomarkers for clinical diagnosis and treatment monitoring [5]. In this study, we focus on two key types of eye movements, saccades and fixations.

 **Saccades**  are rapid movements of the eyes that shift the centre of gaze from one point to another in the visual field [6]. They typically occur in durations between 20 and 300 milliseconds with longer saccades reaching velocities up to 700° per second [7]. This mechanism allows us to efficiently scan our environment and bring objects of interest onto the fovea for sharp, detailed vision. During saccades, visual perception is momentarily suppressed to prevent blurring, ensuring stable and continuous vision as the eyes rapidly reorient.
 **Fixations**  are periods of relative stability, holding the gaze on a single location to allow for detailed visual processing. Fixations serve as the primary means for gathering detailed visual information, as they allow the fovea to gather detailed visual information and focus on a specific point. They typically last between 50 and 600 ms, with longer durations often reflecting increased cognitive load or attentional engagement [8]. The ability to maintain stable fixation is essential for tasks requiring fine high-resolution visual interpretation and sustained attention.

Existing research has primarily focused on tasks such as gaze estimation and eye tracking [9], ignoring the classification of fixations and saccades. Neuromorphic Sensors (event cameras) and biologically inspired models such as Spiking Neural Networks offer promising solutions. However, there is no publicly available event-based dataset that contains precise temporal annotations for fixation and saccade sequences hindering development and evaluation. To address this gap, we introduce event data as a new modality for eye movement classification, contribute manual annotations to the EV-Eye dataset, and propose an SNN model tailored for fixation and saccade recognition. This work lays the foundation for future event-driven research in fine-grained eye movement analysis and cognitive modeling.

The contributions of this paper are as follows:

1. A comprehensive manual annotation of the publicly available EV-Eye dataset, specifically segmenting the data into sequences of saccades and fixations using both event streams and grayscale near-eye images, ensuring the dataset is well prepared for future research.
2. A benchmarking study across five established networks, including SpikingVGG11, 13 & 16, SpikingSqueezenet, and SpikingDenseNet, evaluating robustness and efficacy.
3. Spiking-ConvNet for classification of fixations and saccades on the annotated EV-Eye dataset, specifically designed to leverage the sparse, temporal nature of event-based data. Further, to study the effect of temporal granularity, the Spiking-ConvNet is trained and tested across accumulation windows ranging from 20 ms to 200 ms.
4. A computational complexity analysis comparing the proposed SNN with conventional ANN models, highlighting substantial reductions in operations and demonstrating the efficiency of SNNs for low-power, high-speed eye movement classification.

**Figure 1:**
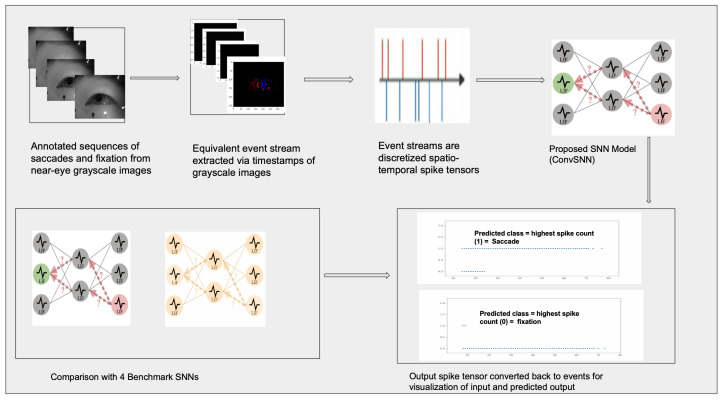
Overview of the proposed eye tracking method. We conduct a cross-evaluator annotation using the EV-Eye dataset to generate sequences of event streams representing fixations and saccades. The resulting event streams are discretized into spatio-temporal spike tensors which are used to train the proposed convolutional Spiking Neural Network (Spiking-ConvNet). Spiking-ConvNet is then compared against four benchmark SNN architectures trained on event-based datasets.

An overview of the proposed methodology is illustrated in Figure 1. The remainder of this section gives background information on eye movement analysis, event cameras, and Spiking Neural Networks. The rest of this paper is structured as follows: Section 2 reviews the history of eye movement classification and highlights the potential of event-based methodologies. Section 3 provides an in-depth overview of SNNs, introduces the proposed model and details the dataset and annotation protocol. Section 4 and Section 5 present the experimental setup and results, including benchmarking evaluations. Finally, Section 7 concludes with an analysis of the findings, a brief discussion of future directions, and closing remarks.

### 1.1. Advancements and Limitations in Eye Movement Analysis Technologies

Eye movement classification and tracking have seen substantial advancements through the expansion of data modalities and the development of increasingly sophisticated algorithms. The classification of fixations and saccades in particular has benefited from the use of multiple modalities, each offering unique strengths but also presenting distinct challenges constrained by trade-offs involving invasiveness, temporal and spatial resolution, and robustness under real-world conditions [10].

Video-Based Eye Trackers are widely used due to their non-invasiveness and high spatial resolution. However, they rely on frame-based imaging that introduces motion blur and temporal aliasing when capturing rapid eye movements such as saccades. This can lead to inaccuracies in onset and offset detection, especially under variable lighting conditions and in high-speed gaze shifts [11]. To address limitations in temporal precision, Scleral Search Coils provide exceptional temporal precision, particularly in controlled laboratory settings [12]. Despite this, they are invasive, requiring the placement of a contact lens with an embedded coil, making them unsuitable for naturalistic clinical applications outside controlled environments.

As a less invasive alternative, Electro-oculography (EOG) provides an alternative by measuring the corneo-retinal potential [13,14]. While this approach is robust to head movements and can be used in mobile scenarios, it suffers from drift, low spatial resolution, and susceptibility to muscle artifacts and electrical noise, which complicates precise eye movement classification. For studies requiring simultaneous eye movement and brain imaging, MRI-compatible eye trackers have been explored for concurrent eye movement and neuroimaging studies [15]. However, these systems are constrained by the MRI environment, often resulting in reduced spatial and temporal resolution, and are limited to specific experimental setups.

In recent years, wearable eye-tracking devices like the Tobii eye tracking glasses have gained popularity for their portability and applicability in real-world settings [16,17]. Nevertheless, these devices often struggle with calibration drift, occlusion, and variable performance in different lighting conditions. Moreover, their frame-based nature limits their ability to capture the fastest eye movements without missed or blurred data.

### 1.2. Event Cameras (ECs)

Given the limitations of conventional eye-tracking modalities, particularly in capturing rapid gaze dynamics under naturalistic conditions, event cameras (ECs) have emerged as a promising alternative. These sensors offer several advantages over traditional approaches, especially in addressing challenges related to temporal resolution, data quality, and real-time responsiveness [18]. ECs record visual changes asynchronously, enabling microsecond-level temporal resolution and minimal latency, which are critical for accurately capturing fast eye movements such as saccades. Their unique operating principle makes them well suited for fixation and saccade classification in eye movement research. Unlike frame-based cameras, ECs operate at the pixel level, detecting and reporting changes in light intensity independently and asynchronously. The resulting data stream consists of discrete “events”, each represented as a tuple:

(1) $$e_{i}=\left(x_{i},y_{i},t_{i},p_{i}\right)$$

where

- $x_{i},y_{i}$ are the spatial coordinates of the event,
- $t_{i}$ is the timestamp of the event,
- $p_{i}\in \left\{-1,+1\right\}$ is the polarity, indicating whether the change in intensity is positive or negative.

An event is generated at pixel (x,y) when the change in logarithmic brightness L(x,y,t) exceeds a predefined contrast threshold θ:

(2) $$\left|L(x,y,t)-L(x,y,t-\Delta t)\right|\geq \theta$$

The brightness is typically modeled as

(3) L(x,y,t)=logI(x,y,t)

where I(x,y,t) is the intensity at location (x,y) and time *t*. The polarity is then defined by

(4) $$p_{i}=\left\{\begin{array}{cc} +1 & \mathrm{if}\;L\left(x_{i},y_{i},t_{i}\right)-L\left(x_{i},y_{i},t_{i}-\Delta t\right)\geq \theta \\ -1 & \mathrm{if}\;L\left(x_{i},y_{i},t_{i}\right)-L\left(x_{i},y_{i},t_{i}-\Delta t\right)\leq -\theta \end{array}\right.$$

This results in high temporal resolution, typically in the microsecond range, allowing for precise capture of rapid eye movements such as saccades. The sparse output of event cameras, where only changing pixels generate data, reduces redundancy and computational load, enabling efficient processing for real-time classification. Additionally, their high dynamic range allows for operation in varying lighting conditions, which is beneficial for eye tracking in diverse environments. The low latency and low power consumption of event cameras further contribute to their suitability for wearable eye tracking devices used in saccade and fixation studies.

### 1.3. Spiking Neural Networks

Complementing this hardware advancement, Spiking Neural Networks (SNNs) have been identified as an ideal match for use with the data from ECs. SNNs are artificial neural networks with a biologically inspired computational framework capable of processing temporally rich event streams [19,20]. Operating through sparse, spike-based communication, SNNs are inherently well matched to the asynchronous nature of event-based data, offering potential in the use of event data directly without the need for a representation of data. SNNs diverge from the continuous activation paradigm of traditional Artificial Neural Networks (ANNs). Unlike ANNs, which rely on continuous valued outputs derived from activation functions (e.g., sigmoid or ReLU), SNNs operate on discrete events known as *spikes*. Each neuron in an SNN integrates its membrane potential, $V_{m}\left(t\right)$, over time and emits a spike at time $t_{s}$ when $V_{m}\left(t\right)\geq \theta$, where θ is the firing threshold.

In contrast to Artificial Neural Networks (ANNs), where information is represented by the average firing rate of neurons over a time window, Spiking Neural Networks (SNNs) employ *temporal coding*, in which the precise timing of individual spikes carries information. This temporal precision enables SNNs to capture fine-grained temporal dynamics, making them particularly well suited for processing data from ECs. Unlike conventional frame-based cameras, ECs asynchronously emit spikes in response to changes in illumination, ΔI(t)>ϵ, producing sparse yet temporally accurate data streams.

This synergy between the asynchronous, sparse nature of EC outputs and the spike-driven computation in SNNs enables ultra-low-latency processing, crucial for real-time classification of rapid eye movements like saccades and fixations. Moreover, SNNs employ learning mechanisms such as Spike-Timing Dependent Plasticity (STDP) [21], where synaptic weight updates, Δw, depend on the relative timing of pre and post-synaptic spikes:

(5) $$\Delta w=\left\{\begin{array}{cc} A_{+}\cdot e^{-\left(t_{\mathrm{post}}-t_{\mathrm{pre}}\right)/\tau_{+}}, & \mathrm{if}\;t_{\mathrm{post}}>t_{\mathrm{pre}}\\ -A_{-}\cdot e^{-\left(t_{\mathrm{pre}}-t_{\mathrm{post}}\right)/\tau_{-}}, & \mathrm{if}\;t_{\mathrm{pre}}>t_{\mathrm{post}} \end{array}\right.$$

This biologically inspired adaptation allows the network to learn temporal patterns critical for distinguishing between different types of eye movements. Furthermore, both SNNs and ECs are characterized by low power consumption and high adaptability, making them ideal for integration into portable eye-tracking devices. SNNs dynamically adjust their computational load in response to the density of events generated by ECs, maintaining efficient bandwidth usage and robust classification performance even under varying motion speeds or degraded lighting conditions. Their resilience to sparse and noisy input, along with their biologically plausible structure, contributes to accurate, interpretable modeling of human visual behavior.

## 2. Related Works

In this section, we present a comprehensive review of existing methodologies for fixation and saccade classification, tracing the evolution from traditional threshold-based algorithms to data-driven machine learning approaches. We highlight the emerging role of event-based vision and Spiking Neural Networks (SNNs) in addressing the limitations of conventional systems, particularly in terms of temporal precision, energy efficiency, and biological plausibility.

### 2.1. Eye Movement Classification Technologies

Eye movement analysis has been a critical task in understanding various mechanisms of the human brain and its application to domains such as cognition, clinical neuroscience, and human–computer interaction [22]. Different eye behaviors are associated with distinct cognitive and physiological functions. For instance, blinking frequency has been linked to attention modulation and fatigue detection [23], while gaze dynamics are widely used to facilitate adaptive human–computer interfaces [24]. Moreover, pupil dilation and microsaccades have been studied as indicators of arousal and decision uncertainty [25].

Saccades and fixations in particular play a pivotal role in daily tasks such as reading, where a typical fixation lasts between 200 and 250 milliseconds and saccades span approximately 20–40 ms, covering 7–9 characters per movement [26]. These eye movements have also been associated with decision-making processes and are increasingly studied as biomarkers for psychiatric disorders such as depression, bipolar disorder (BD), and anxiety disorder (AD) [27]. Their mechanism is altered in neurodegenerative diseases, including Alzheimer’s and Parkinson’s disease, where oculomotor abnormalities such as hypometric saccades, increased latency, and fixation instability have been observed [28]. In the context of Parkinson’s disease, these metrics are gaining attention as non-invasive, quantifiable biomarkers for early detection [29].

Early approaches to fixation and saccade classification predominantly relied on threshold-based algorithms, such as Identification by Dispersion-Threshold (I-DT) and Velocity-Threshold Identification (I-VT) [30,31]. These methods segment eye movements based on spatial dispersion and velocity thresholds. While computationally efficient and straightforward to implement, they are highly sensitive to noise and require manual calibration, which limits their generalization across participants and recording setups [32,33,34].

To overcome the constraints of fixed threshold algorithms, more recent studies adopted machine learning techniques capable of learning adaptive patterns from eye tracking data [10]. Random Forests and Convolutional Neural Networks (CNNs) have demonstrated improved accuracy and robustness. For instance, McCarty et al. [35] showed that Random Forests outperform Logistic Regression and K-Nearest Neighbors, while Wang et al. [36] proposed a cascade forest model to address class imbalance. Birawo et al. [10] further validated the superiority of CNNs and Random Forests over traditional methods. More advanced techniques have emerged, including adaptive decision trees [21], Radial Basis Function Neural Networks, and Markov Chains for unsupervised segmentation [37]. Additionally, cross-modal approaches such as integrating EEG signals with neural networks [38] or applying CNN-LSTM hybrids to fixation heatmaps [39] have broadened the analytical scope of research.

With the emergence of efficient eye tracking hardware, recent research has increasingly leveraged data from such modalities, particularly high-resolution gaze signals from commercial trackers (e.g., Tobii [40]) and webcam-based recordings [11]. Yet, these modalities, while providing more accurate data, remain susceptible to several limitations. Noise artifacts, motion blur, variable lighting conditions, and low frame rates can significantly degrade signal quality. Moreover, head pose variation, occlusion, and inter-subject variability introduce challenges in generalization and robustness across diverse populations [9]. These constraints underscore the need for further algorithmic refinement and hybrid sensor fusion strategies to enhance resilience and scalability in real-world applications.

### 2.2. Event Cameras and Spiking Neural Networks

In contrast to traditional eye-tracking modalities and algorithms, event cameras offer microsecond-level resolution and asynchronous data capture, rendering them particularly well suited for analyzing rapid eye movements [9]. Their attributes, such as high temporal resolution, high latency, and low dynamic range, make them especially effective for capturing rapid eye movements, including saccades and fixations, which frequently occur on millisecond timescales [41]. Over the past decade, numerous studies have emerged within the event-based vision domain, with several works demonstrating the potential of event-based vision for robust eye movement analysis and temporal feature extraction techniques [9]. Applications range from gaze tracking [42,43], pupil tracking [44,45], and blink detection [46] to eye tracking [36,47]. However, none of these studies have directly addressed the classification between saccades and fixations.

In recent research, there has been a dual focus on advancing algorithms and exploring the potential of Spiking Neural Networks (SNNs) as a supplementary approach for handling event data [48,49]. SNNs, which are modeled after biological neurons, exhibit a natural compatibility with event-based data due to their sparse, asynchronous processing and energy-efficient design [48,50,51]. Studies have shown that SNNs are capable of effectively capturing detailed temporal dynamics in applications such as object detection and scene understanding [52]. The spike-based representation of SNNs offers a computationally efficient and biologically plausible alternative to traditional deep learning models.

Although SNNs have been explored in eye-tracking tasks, their direct application to fixation and saccade classification remains under-explored. To the best of our knowledge, this study is the first to apply SNNs directly to event-based eye movement classification. To address limitations inherent in conventional eye-tracking modalities and algorithms, our research fills a critical gap by leveraging event data and SNNs for a more robust temporal analysis. Moreover, only a few prior studies [53,54,55,56] have investigated end-to-end SNN architectures operating on raw event streams for eye movement classification. Our study fills this gap by contributing to neuromorphic computing and highlighting the role of this field in advancing eye movement research.

## 3. Methods

In this section, we provide an overview of how event-based data are represented for processing by the Spiking Neural Networks (SNNs) followed by a detailed description of the network architectures employed in this study.

### 3.1. Event Representation

The dataset generated by event cameras (ECs) differs fundamentally from conventional CCD/CMOS cameras. Thus, there is the need to convert raw event streams into a format that can be utilized by neural networks. Rather than accumulating events into representations like frames or time surface as seen in prior works [57], we discretize the asynchronous stream into spatio-temporal spike tensors, preserving its event-driven nature and aligning with the spike-based computation of SNNs. This also plays a role in real-time efficiency. To ensure consistent temporal segmentation, we adopt a fixed-window binning strategy, as intervals between fixations and saccades with minimal motion may yield low event density. This encoding follows a *temporal coding* scheme, where spike timing conveys information about the signal. Unlike latency-based encoders such as Time-To-First-Spike (TTFS) [58], which restrict neurons to a single spike, this method allows multiple spikes per neuron across time bins, capturing richer temporal dynamics.

Each event is represented as (x,y,p,t), where x,y denote spatial coordinates, p∈{0,1} indicates polarity (ON/OFF), and *t* is the timestamp. Events are binned into a four-dimensional tensor $S\in R^{C\times H\times W\times T}$, where C=2 corresponds to polarity channels and $T=\frac{L}{\Delta t}$ denotes the number of temporal bins. Given an event set $E={\left\{\left(x_{i},y_{i},p_{i},t_{i}\right)\right\}}_{i=1}^{N}$, each event is assigned to a temporal bin $b_{i}=\left\lfloor \frac{t_{i}}{\Delta t}\right\rfloor$ and updated as

$$S[p_{i},y_{i},x_{i},b_{i}]=1.$$

The corresponding spike rate is defined as

$$r_{x,y,p}=\frac{1}{T}\sum_{t=0}^{T-1}S\left[p,y,x,t\right].$$

This representation preserves both spatial structure and temporal spike density, enabling the network to distinguish between saccades and fixations based on their characteristic motion dynamics.

### 3.2. Spiking Neural Networks and Neuron Dynamics

A fundamental component of any SNN is the neuron model, which governs the evolution of membrane potential and spike generation over time. Among the various models proposed, the Leaky Integrate-and-Fire (LIF) neuron remains one of the most widely adopted due to its balance between biological plausibility and computational efficiency [59]. The LIF neuron integrates incoming current and leaks over time, emitting a spike when the membrane potential exceeds a threshold. In this study, we adopt the Current-Based Leaky Integrate-and-Fire (CUBA-LIF) neuron model, which extends the conventional LIF formulation by decoupling synaptic current integration from membrane potential decay [60]. This architectural separation enables more flexible temporal filtering and enhances biological plausibility, particularly in modeling asynchronous event-driven dynamics observed in neuromorphic systems. The membrane potential update in the presence of recurrent connections is often expressed as

(6) $$U_{i,t}=\beta U_{i,t-1}+V_{i}^{\left(\mathrm{res}\right)}S_{i,t-1}+I_{i,t}^{\mathrm{in}}-RU_{i,t},$$

where $V_{i}^{\left(\mathrm{res}\right)}$ represents a linear transformation applied to the previous spike $S_{i,t-1}$ and *R* is the reset term. This formulation enhances temporal dynamics through feedback, and when the reset mechanism is omitted (i.e., “none”), the equation simplifies by removing the $RU_{i,t}$ term. In our implementation, we adopt the discrete-time approximation of CUBA-LIF dynamics using the Lava framework. The synaptic current u[t] and membrane potential v[t] evolve according to

(7) $$\begin{array}{cc} u[t] & =\left(1-\alpha_{u}\right)\cdot u\left[t-1\right]+x\left[t\right], \end{array}$$

(8) $$\begin{array}{cc} v[t] & =\left(1-\alpha_{v}\right)\cdot v\left[t-1\right]+u\left[t\right]+b, \end{array}$$

where $\alpha_{u}$ and $\alpha_{v}$ are decay factors derived from the synaptic and membrane time constants, respectively. A spike is emitted when the membrane potential exceeds a threshold ϑ, and the voltage is reset:

(9) $$\begin{array}{cc} s[t] & =\Theta (v[t]-\vartheta ), \end{array}$$

(10) $$\begin{array}{cc} v[t] & =v[t]\cdot (1-s[t]). \end{array}$$

**Figure 2:**
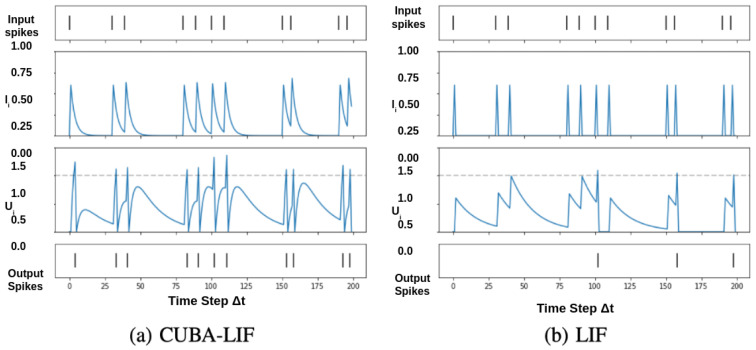
Temporal dynamics across neuron models: (**a**) CUBA-LIF and (**b**) LIF. Each row shows input spikes, synaptic current ($I_{j}$), membrane potential ($U_{j}$), and output spikes over time. The dashed line in $U_{j}$ indicates the spike threshold [60].

This two-stage dynamic introduces persistent state variables that enable temporal filtering and refractory behavior. To further illustrate the temporal evolution and comparative behavior of the CUBA-LIF neuron, Figure 2 presents a side-by-side visualization with LIF under identical input conditions.

By tuning the ratio $\tau_{\mathrm{syn}}/\tau_{\mathrm{mem}}$, we can modulate the model’s responsiveness and integration properties. For instance, a small $\tau_{\mathrm{mem}}$ increases leakage, requiring stronger inputs to trigger spikes, while a large $\tau_{\mathrm{syn}}$ prolongs synaptic integration, facilitating temporal summation.

### 3.3. Model Architecture

**Figure 3:**
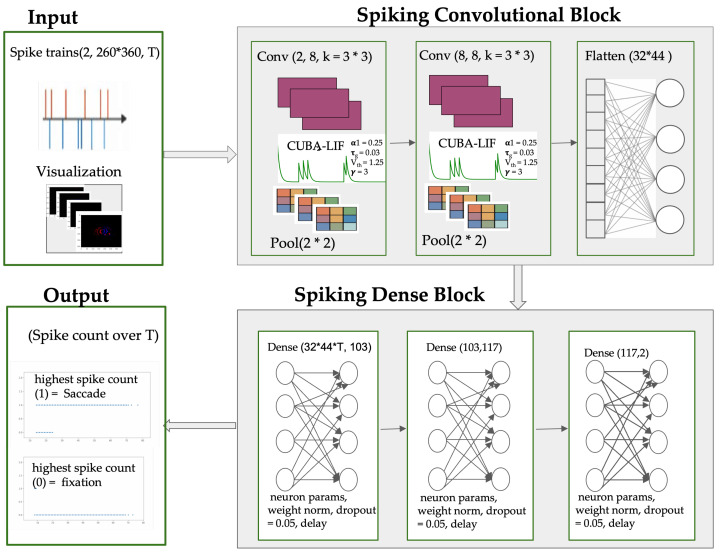
Overview of the proposed Spiking-ConvNet architecture: event streams are fed directly into the SNN through a spike-based encoding that preserves the temporal structure of the input. Convolutional layers extract spatiotemporal features associated with saccadic and fixational eye movements, after which the resulting feature maps are flattened and passed to the fully connected layers. Dropout and synaptic delays are then incorporated to enhance generalisation and support temporal integration to produces class-specific spike-rate outputs.

To better capture the spatio-temporal dynamics of saccades and fixations, we implemented an SNN architecture comprising convolutional layers, which we call Spiking-ConvNet. An overview of this architecture is illustrated in Figure 3. This hybrid architecture, using the CUBA-LIF, combines spatial feature extraction with temporal integration, preserving the precision of spike-based processing while enhancing spatial encoding. This architecture is inspired by standard SNN implementations in *Lava* [61] and *SLAYER* [62] for benchmarking on N-MNIST [63].

The architectural choices of the proposed Spiking-ConvNet are heavily informed by the need to efficiently utilize the temporal structure in event-based eye movement data while enabling a reduced computational cost and maintaining strong performance. While the basic LIF neuron provides a useful baseline, it does not fully leverage the rich temporal information present in event streams. Incorporating membrane and synaptic time constants through the CUBA-LIF model introduces a more precise and biologically grounded integration of incoming events. This enhanced temporal filtering is particularly important for modeling eye movements such as saccades, microsaccades, and fixational jitter, where event density fluctuates rapidly. Synaptic currents act as temporal filters that allow neurons to respond to motion patterns rather than isolated spikes, improving sensitivity to saccade duration and reducing spurious activations caused by small, rapid eye movements. The resulting smoothing effect stabilizes firing rates across transitions between fixations and saccades, enabling the architecture to achieve strong performance with fewer parameters and improved robustness under variable input statistics.

The input to the Spiking-ConvNet has shape (B,2×260×360,T) where *B* is the batch size and *T* denotes the number of time bins defined by the temporal resolution. The input encodes ON/OFF polarity across the spatial resolution of the event stream. A first convolutional layer with 3×3 kernels and Stride 2 extracts local spatio-temporal features while reducing dimensionality, followed by a max-pooling layer with Stride 2 to suppress redundancy. A second convolutional layer with 3×3 kernels and Stride 1 further refines feature maps, again followed by pooling to downsample the representation. The resulting feature maps are flattened into a dense vector, which is processed by two fully connected layers of CUBA-LIF neurons (sizes 11,264→103 and 103→117). These dense layers incorporate dropout (p=0.05), delay-based synapses, and weight normalization to enhance generalization and temporal learning. The final output layer maps 117 neurons to 2 spiking neurons, enabling binary classification. Surrogate gradient descent is employed for training, ensuring differentiability of spike-based dynamics. The full layer-wise configuration is detailed in Table 1.

Beyond the neuron model, the convolutional connectivity pattern is well suited to the spatiotemporal structure of eye movement data. The local receptive fields capture fine grained motion, while hierarchical feature extraction supports and adds temporal context across multiple scales. This aligns naturally with the sparse, asynchronous nature of event streams, allowing the network to process only the spatiotemporal regions where activity occurs.

The use of a surrogate-gradient backpropagation learning rule further enhances this architecture by enabling effective temporal assignment in the presence of discontinuous spike events. It allows the network to learn precise spike-timing relationships and adapt synaptic dynamics to the statistics of eye movement patterns. Together, these design choices leverage the unique properties of SNNs such as temporal coding, reduction in computation due to sparsity and low power inference.

To decode the network’s prediction, we adopt a spike-rate-based classifier, wherein the class label is determined by comparing the average firing rate of the two output neurons over the simulation window. Formally, we let $s_{i}\left[t\right]$ denote the spike output of neuron *i* at time *t*, then the predicted class $\hat{y}$ is given by

$$\hat{y}=\arg\max_{i\in \{0,1\}}\left(\frac{1}{T}\sum_{t=0}^{T-1}s_{i}\left[t\right]\right).$$

During training, surrogate gradient descent optimizes the network parameters, with the spike-rate loss function guiding the output neurons to emit discriminative firing patterns. The decoding method is implemented via SLAYER Rate Predict, which computes the mean spike count per output neuron and returns the class with the highest response.

### 3.4. Datasets

Event-based eye tracking has seen an increase in available datasets used for experimentation over the last decade [9], yet none of these have explicit annotation sequences for saccades and fixations. The earliest relevant dataset by Angelopoulos et al. [64] includes greyscale images with a spatial resolution of 260 × 360 labeled with timestamps and motion coordinates for saccades, fixations, and smooth pursuits. However, when inspected closely, the annotations are inaccurate for some sequences.

The EV-Eye dataset contains greyscale images, with a spatial resolution of 260×260, event streams, and synchronized gaze data from DAVIS346 cameras and Tobii Pro glasses [40], respectively. The availability of raw event streams alongside synchronized RGB data renders it well suited for annotation into saccade and fixation sequences. We curated a subset of EV-Eye by manually annotating saccades and fixations for the left eye of 10 participants. Right-eye sequences were programmatically derived via temporal alignment, given the conjugate nature of binocular eye movements [65].

The classification protocol is inspired by the taxonomy and evaluation of fixation identification algorithms proposed by Salvucci et al. [31], which outlines how spatial and temporal parameters are used to differentiate fixations from saccades in eye tracking data. Fixations were defined as periods where the pupil remained spatially stable for at least 20 ms, typically indicating visual attention. Saccades were identified as transitions between fixations, consistent with the EV-Eye acquisition setup [66]. Annotation was conducted by two annotators, Author 1 and 3, each independently responsible for identifying one class, either saccades or fixations, from the greyscale images of EV-Eye. To ensure accuracy and consistency, each annotation was cross-checked. This dual-role approach enabled cross-validation and facilitated the identification and correction of annotation errors across both movement types, thereby enhancing the reliability of the dataset.

**Figure 4:**
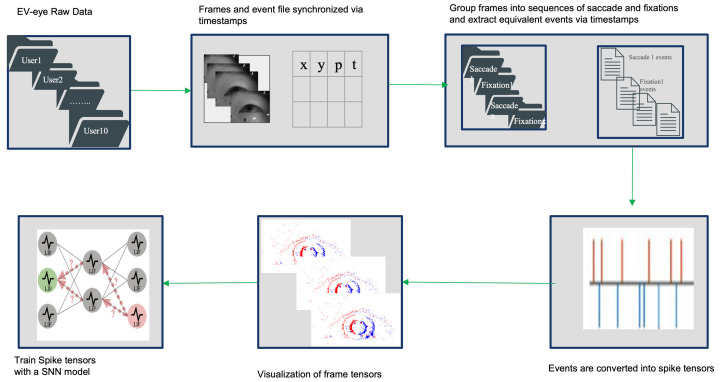
Overview of the annotation procedure within the EV-Eye dataset. The diagram illustrates how annotations are linked to specific sessions and modalities, enabling precise labeling sequences such as fixations and saccades.

Event-based annotations were generated by aligning the timestamps of each annotated RGB sequence with the corresponding event stream segment. This ensured precise temporal correspondence between greyscale and event data. A schematic overview of the annotation pipeline is shown in Figure 4.

This annotation process yielded a total of 1850 saccade sequences and 1326 fixation samples, with the saccade set later downsampled to 1326 for class balance. Upon further preprocessing, 1000 samples of each class were retained as the final dataset. We trained on 80% (Users 1–7) of the data and reserved 20% (Users 8–10) for testing.

**Figure 5:**
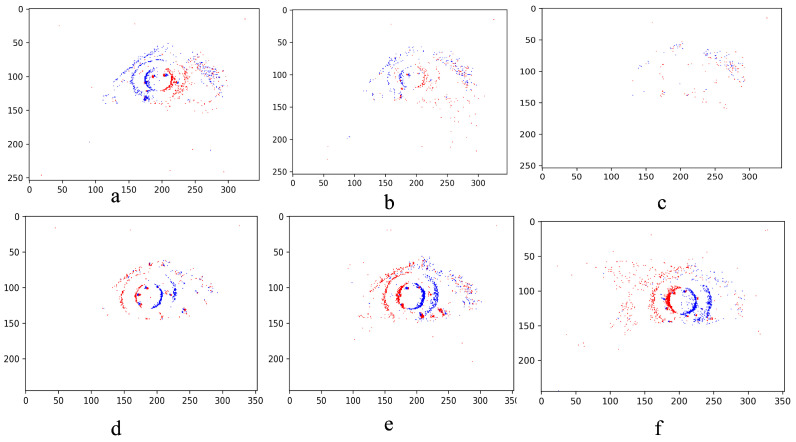
Illustration of frame-based representations of fixation and saccade sequences, with red and blue dots indicating positive and negative event polarities, respectively. The top row (**a**–**c**) illustrates a sequence of fixation frames, while the bottom row (**d**–**f**) illustrates a saccade sequence. Notably, the eye is absent in the last fixation frame which is an example of event generation in fixations, i.e., minimal motion and thus produce fewer events. In the saccade sequence, we see a difference in eye positions as well as lower event density in beginning and landing frames. Although this does not represent the entire sequences, these observations underscore the limitations of frame reconstruction from event data, particularly in the context of eye movement analysis, where temporal precision and motion sensitivity are critical.

Figure 5 illustrates a representative sequence of fixation and saccade from the EV-Eye dataset.

## 4. Experimental Configurations

This section details the training setup, evaluation, and results of the proposed model’s performance, measured against event-based benchmarks.

### 4.1. Training Setup

The SNN architecture implemented using the Lava-DL SLAYER framework [62]. Lava provides a flexible and modular system for constructing event-based SNNs. Training was conducted using SLAYER’s backpropagation-compatible framework with graded spikes, enabling differentiable learning. SLAYER’s utilities support event-based I/O, visualization, and logging. Neuron parameters were learnable, with batch normalization and dropout applied for stability and generalization. The model was trained using spike rate loss over 100 epochs (batch size 8, learning rate 0.01, Adam Optimizer), with Cuba-LIF neurons and surrogate gradient descent. All models were trained on an NVIDIA GeForce RTX 2080 Ti GPU.

### 4.2. Evaluation (Loss Function)

The SLAYER framework provides predefined loss modules such as *SpikeTime*, *SpikeRate*, *SpikeMax* with delineated applications. For this implementation, *SpikeRate* loss is adopted due to the absence of ground-truth target spike trains. The objective enforces high firing rates ($r_{\mathrm{true}}$) for the true class and suppresses spiking ($r_{\mathrm{false}}$) in non-target neurons. We let $1\left[l\right]\in {0,1}^{C}$ denote the one-hot encoded label vector for *C* classes. The target rate vector $\hat{r}\in R^{C}$ is formulated as

(11) $$\hat{r}=r_{\mathrm{true}}\cdot 1\left[l\right]+r_{\mathrm{false}}\cdot \left(1-1[l]\right)$$

where 1 is a ones vector. The loss function computes the mean squared error between the empirical firing rate $\frac{1}{T}\int_{T}s\left(t\right)dt$ and target rates:

(12) $$L=\frac{1}{2}\left|\frac{1}{T}\int_{T}s\left(t\right)dt-\hat{r}\right|2^{2}=\frac{1}{2}{\left(\frac{1}{T}\int Ts\left(t\right)dt-\hat{r}\right)}^{⊤}1$$

SpikeRate prevents the need for exact spike timing supervision while maintaining interpretability through rate modulation. By assigning $r_{\mathrm{true}}\gg r_{\mathrm{false}}$, the model learns to associate specific neurons with class-specific activation patterns, approximating decision boundaries via rate coding. This approach proves effective when target spatiotemporal characteristics are underspecified. Compared to other loss functions available for SNNs, the SpikeRate loss offers a practical balance in performance. Unlike SpikeTime loss, which requires precise supervision of spike timing and is thus more challenging to optimize, SpikeRate loss only needs the desired firing rates for each output neuron, while advanced losses like SpikeMax or enhanced counting losses can provide additional benefits, such as improved gradient flow or parameter-free operation.

## 5. Results

This section presents a comprehensive evaluation of our approach. First, we evaluate the performance of the proposed SNN *Spiking-ConvNet*, positioning among benchmark SNN architectures such as SpikingDensenet and SpikingVGG variants [67]. We achieve this in terms of classification accuracy, loss, precision, and recall. Following this, we explore the performance of the proposed model across varying temporal resolutions to analyze its sensitivity to time-based granularity. Finally, we assess computational efficiency by comparing the energy and resource demands of the proposed SNN against standard artificial neural networks (ANNs), demonstrating the advantages of SNNs in low-power scenarios.

### 5.1. Comparison with State-of-the-Art SNN Models

We evaluate the performance of the proposed Spiking-ConvNet architecture in the context of other SNN models trained on event-based data by conducting a comparative analysis against several widely adopted models that serve as neuromorphic equivalents to conventional deep learning benchmarks. Specifically, we include SpikingVGG11, SpikingVGG13, SpikingVGG16, SpikingDenseNet, and a lightweight model, SpikingSqueezeNet. These models are implemented by Cordone et al. in their study *Object Detection with Spiking Neural Networks on Automotive Event Data* [67].

Spiking VGG variants typically rely on deep, sequential convolutional stacks with uniform kernels, which inflate parameter counts and generate substantial spike activity. In contrast, Spiking-ConvNet reduces depth and incorporates selective feature extraction stages (spiking convolutional block), limiting redundant spiking and improving computational efficiency without sacrificing representational power. While the Spiking DenseNet model promotes feature reuse through dense inter-layer connectivity, this design introduces heavy memory traffic and elevated spike accumulation. This may pose challenges for neuromorphic hardware with constrained bandwidth.

The proposed SNN avoids dense skip connections and instead adopts streamlined pathways that preserve essential features while reducing memory access overhead. Spiking SqueezeNet architectures achieve parameter compression through bottleneck “fire” modules; however, these reductions can restrict the temporal precision needed for event-driven tasks. Inspired by these challenges, the proposed SNN priorities real-time use, leading to deliberate trade-off between depth, connectivity, and spike efficiency, making it better suited for event-based eye movements tasks and neuromorphic deployment.

While our task is a very different application domain, their inclusion provides a diverse and representative benchmark suite, given their demonstrated performance across neuromorphic datasets such as NCaltech and NCars, as well as their architectural variety in depth and parametrization. To ensure a fair and consistent evaluation, all benchmark models were applied to the annotated subset of the EV-Eye dataset under identical preprocessing and accumulation conditions. Table 2 summarizes the performance metrics, including accuracy, precision, recall, and F1-score, for each model. This comparative analysis enables a direct assessment of Spiking-ConvNet’s efficacy relative to established spiking architectures, while controlling for dataset-specific variability.

While the proposed Spiking-ConvNet does not surpass the benchmark models in conventional classification metrics, its performance remains contextually strong when considering architectural and representational differences. Unlike benchmark models that rely on voxelized event representations, Spiking-ConvNet processes raw spiked event streams directly, allowing the use of the inherently sparse and temporally fine-grained event-based data. This design choice leverages spike-based computation to reduce redundancy and promote computational and energy efficiency. By eliminating the need for frame-based preprocessing and intermediate representations, Spiking-ConvNet minimizes computational overhead and latency, enabling more efficient real-time inference suitable for neuromorphic hardware.

Despite operating under these constraints, Spiking-ConvNet achieves an accuracy of 93.06%, with precision and recall exceeding 92%, demonstrating reliable detection of saccade and fixation events. Notably, it is the first model to explicitly classify saccades and fixations from event-based eye movement data, whereas existing benchmarks focus on broader motion or object recognition tasks; rendering direct comparisons less accurate.

While the proposed architecture prioritizes parameter efficiency, we acknowledge that this design introduces several trade-offs. A key factor influencing performance is the choice of temporal resolution which is discussed in detail in the following section. The reduced parameterization of the model also introduces a degree of sensitivity to training hyperparameters. Achieving stable convergence may require more careful tuning of learning rates, thresholds, and regularization strategies compared to larger, more redundant architectures. These trade-offs are outweighed by the substantial gains in efficiency, making the architecture particularly suitable for real-time or resource-constrained neuromorphic applications. Future work may further explore how alternative neuron models or adaptive temporal windows influence real-time performance and inference robustness.

### 5.2. Performance Across Temporal Resolutions

Given the asynchronous nature of event-based data, a common approach to pre-processing these data is typically achieved through event accumulation, either by aggregating a fixed number of events or by discretizing time into uniform windows. In this work, we adopt a time-window-based accumulation strategy, where the duration of the window directly determines the temporal granularity of the input. The choice of accumulation window size is critical: shorter windows preserve fine temporal detail but may yield sparse input frames, while longer windows increase event density at the cost of temporal precision. This trade-off is particularly relevant for saccadic eye movements, which typically span durations between 20 ms and 200 ms. Capturing the rapid onset and offset of saccades requires sufficient temporal resolution to preserve motion dynamics, yet overly fine resolutions may introduce noise, reduce event density, and increase computational overhead.

To evaluate the impact of temporal granularity on model performance, we trained and tested the proposed Spiking-ConvNet across a range of accumulation windows from 10 ms to 200 ms. This analysis serves two purposes: to assess the robustness of the model under varying temporal conditions and to identify an optimal resolution that balances classification accuracy, computational efficiency, and real-time feasibility. The results, summarized in Table 3, reveal that intermediate resolutions (e.g., 33 ms) offer a favorable trade-off, preserving sufficient temporal detail while maintaining stable performance and frame rates.

Accuracy improves significantly from 94.16% at 10 ms (100FPS) to 96.51% at 20 ms (50 FPS), illustrating how extremely short accumulation windows can fail to capture sufficient spatiotemporal structure for effective convolutional processing. Beyond 20 ms, performance stabilizes, with the model maintaining over 94.96% accuracy at 33 ms (30 FPS) and reaching 96.70% at 100 ms (10 FPS). The highest accuracy of 97.67% is observed at 200 ms (5 FPS), indicating that moderate accumulation windows offer an optimal balance between temporal resolution and classification reliability. Interestingly, while the F1 score peaks at 200 ms (5 FPS) with 0.9767, the accuracy slightly drops to 97.67%, suggesting that longer windows may introduce temporal blurring or instability in fast transitions such as saccades.

We included the effective frame rate (FPS) associated with each accumulation window to contextualize real-time feasibility. Higher FPS corresponds to shorter accumulation windows, enabling faster inference and more responsive interaction, further highlighting the usability of event-based data for this task in real-time settings. However, shorter windows (e.g., 10 ms) increase computational load and may reduce event density per frame, while longer windows (e.g, 200 ms) aggregate more events but risk losing temporal precision. Despite this trade-off, our model remained lightweight and efficient, successfully training even at high-resolution windows like 200 ms, demonstrating its scalability and robustness across temporal settings.

**Figure 6:**
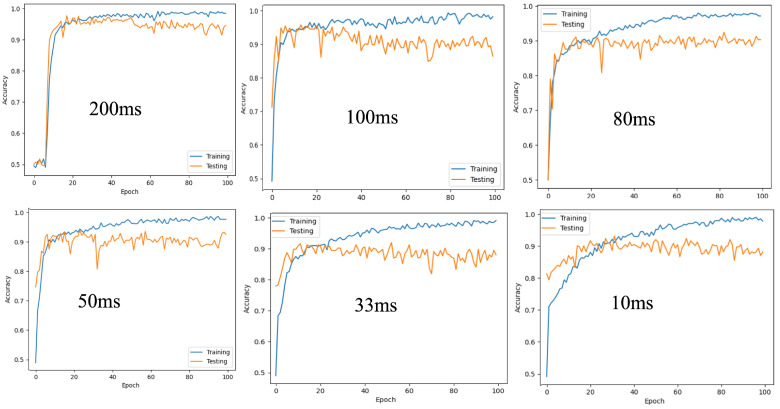
Training and loss graphs for proposed model (Spiking-ConvNet) at different temporal resolutions.

Figure 6 provides a visual insight into how the proposed model adapts to finer-grained temporal inputs. Spiking-ConvNet exhibits stable and consistent training behavior across temporal resolutions from 200 ms and below, with notably lower loss values and smoother convergence, especially at 50 ms and 33 ms. This stability can be attributed to the ability of convolutional layers to capture hierarchical spatiotemporal patterns, making Spiking-ConvNet more resilient to noise and jitter in event timing. The model starts to decrease in performance with no stable training until later epochs for 10 ms. This can be attributed to the relatively small amount of events within this temporal duration, rendering convolutional layers with little features to extract.

It is important to note that the choice of temporal resolution not only affects model performance but also significantly influences training time and computational cost. Longer accumulation windows, such as 200 ms, tend to yield higher classification accuracy due to increased event density per spike train; however, they also result in fewer training samples and longer sequence durations, thereby increasing the time required for each training epoch. In contrast, shorter windows produce more frequent frames, accelerating data throughput but potentially compromising performance due to sparsity. For large-scale datasets, this trade-off necessitates careful optimization, balancing temporal fidelity with computational efficiency.

Additionally, the results presented in Figure 6 reflect early convergence driven by the temporal characteristics of event-based data. A reduction in accumulation window shows how the network reaches stable performance within less epochs, indicating effective extraction of the available information content. This behavior is reinforced by the close alignment of training and validation losses for temporal resolutions above 33 ms, suggesting how sparse spiking activity and synaptic dynamics inherently constrain the model’s effective capacity and mitigate overfitting. Consequently, performance is governed primarily by the temporal information encoded within the accumulation window rather than by extended training duration or network depth. Very short windows provide limited event density, while longer windows introduce temporal redundancy that improves accuracy but yields diminishing returns. This mechanism highlights the model’s convergence behavior as a function of temporal-window selection, and that stable, competitive performance can be achieved even with relatively short windows.

In terms of inference, we observed that training with a 200 ms window required approximately **598.333s per epoch**, with inference per batch averaging **10.18 s**. By comparison, reducing the window to 33 ms decreased training time to **72.71 s per epoch** and inference latency to **8.61 s**, albeit with a modest drop in accuracy. These results highlight the practical deployment trade-off: longer windows improve accuracy but slow down throughput, while shorter windows enable faster training and real-time inference at the cost of reduced event density. Such timing measurements are critical for deployment scenarios, where computational budgets and latency constraints must be balanced against accuracy requirements.

### 5.3. Ablation Studies (Computational Efficiency)

To better understand the contributions of the proposed model compared to conventional algorithms, we performed an ablation study on the computational efficiency of the proposed SNN (Spiking-ConvNet) against computations of an equivalent Artificial Neural Network (ANN). This provided insights into how efficient and computationally beneficial it is to use SNNs and event data for our task as opposed to standard works using RGB and other data modalities. We explored the differences in an assessment of event-driven computation (events and synapses) versus dense multiply-accumulate operations (MACs) and Accumulated Computation (AC). In SNNs, synaptic transmission triggered by spikes requires only an AC operation, which is significantly less energy-intensive than the MAC operations used in ANNs. The total computational cost can be expressed as

(13) $$E\left(F\right)=T\cdot \left(f_{r}\cdot E_{\mathrm{AC}}\cdot O_{\mathrm{AC}}+E_{\mathrm{MAC}}\cdot O_{\mathrm{MAC}}\right)$$

where *T* is the simulation time, $f_{r}$ is the average firing rate, and $O_{\mathrm{AC}}$, $O_{\mathrm{MAC}}$ denote the number of AC and MAC operations, respectively.

Table 4 provides a layer-wise comparison of computational complexity between the proposed Spiking-ConvNet and its equivalent ANN. Overall, the SNN processes **2662.25** spike events across **83,527.26** synaptic operations, whereas the ANN requires over **4.77 million** MAC operations. This corresponds to a reduction of approximately **7×** in total operations, underscoring the efficiency of event-driven computation when exploiting temporal sparsity. The disparity is most pronounced in the early convolutional layers. For example, Layer-1 in the ANN performs 184,728 MACs, while the SNN requires only 367.31 events. Similarly, Layer-2 shows a drop from 3.37 million MACs in the ANN to just 1232.50 events in the SNN. Even in the dense layers, where spike counts decrease further, the SNN still achieves significant savings: Layer-4 reduces computation from 1.16 million MACs to just 7.12 events, and Layer-5 cuts operations from 12,051 MACs to 2.00 events.

These results demonstrate that Spiking-ConvNet consistently achieves two to three orders of magnitude fewer operations per layer while maintaining representational capacity. The total neuron count of 287,366 reflects the architectural scale shared by both models, while the event sparsity of 107.94× demonstrates how infrequently neurons fire in the SNN relative to dense ANN activations. Similarly, synapse sparsity of 57.12× highlights the reduction in effective synaptic operations compared to the full MAC budget of the ANN. With a mean squared error of 0.024825 sq. radians, redundant computation is minimized while the model leverages temporal sparsity to deliver substantial efficiency gains, making it particularly well suited for real-time, low-power neuromorphic applications. This efficiency reinforces how SNN retains accuracy while drastically reducing computational requirements through temporal and structural sparsity and positions Spiking-ConvNet as a promising candidate for energy-constrained and latency-sensitive vision tasks.

## 6. Discussion

A limitation of this work concerns the size of the dataset. While the use of recordings from only ten subjects may be of concern for generalization, event-based data differ fundamentally from RGB imagery. Events are triggered solely by motion and changes in light intensity, resulting in representations largely invariant to appearance-based factors such as skin tone, facial morphology, or illumination. This modality-specific property mitigates these concerns, as the model relies primarily on movement dynamics rather than static visual features. Furthermore, the near-eye acquisition setup inherently reduces the presence of extra cues that could otherwise introduce population-specific biases. We also observed that model performance improves with increased sample availability, including samples initially excluded to create a balanced dataset. Additionally, while the proposed model exhibits a 6% accuracy reduction relative to SpikingVGG16, this trade-off is justified by substantial gains in deployment and real-time suitability. SpikingVGG16’s depth and computational demands make it impractical for embedded and portable clinical systems, whereas the lightweight architecture presented here enables fast, stable inference on neuromorphic hardware with significantly lower energy consumption. These characteristics are essential for continuous monitoring scenarios in which responsiveness, robustness, and power efficiency outweigh marginal improvements in accuracy. While SpikingSqueezeNet contains fewer parameters and achieves accuracy close to that of the proposed Spiking-ConvNet, its bottleneck-centric design restricts temporal expressiveness and interacts suboptimally with sparse event-based inputs, leading to reduced robustness and less efficient spike processing. In contrast, Spiking-ConvNet achieves a substantial reduction in model complexity, requiring only 1.1 million parameters—far fewer than SpikingVGG16 (14.7 M) or SpikingDenseNet (6.9 M). This compactness, combined with stable performance and a design tailored to the dynamics of eye movement events, underscores its suitability for resource-constrained neuromorphic platforms. The architecture demonstrates that convolutional layers can effectively exploit sparse temporal computation without sacrificing representational capacity, offering an efficient and interpretable solution for fine-grained event-based classification. Building on these limitations, future work will extend this baseline beyond categorical classification toward the detection and characterization of saccades, fixations, and additional oculomotor parameters. Expanding the framework to capture richer temporal and kinematic features will enable a more comprehensive representation of eye-movement behavior. Ultimately, future projects will be aimed toward the development of a low-resource, event-driven biomarker capable of identifying early indicators of neurodegenerative disease, leveraging the unique advantages of neuromorphic sensing to support accessible and clinically meaningful screening tools.

## 7. Conclusions

In conclusion, detecting rapid eye movements such as saccades remains challenging due to issues with data quality, diverse sources, and limitations of conventional algorithms. The framework proposed introduces the combination of event camera modality along with the processing capabilities of SNNs to classify near-eye movements into saccades and fixations. Using the EV-Eye dataset, we annotated eye tracking data into sequences of fixations and saccades that were used to train and test the SNN model. The proposed convolutional-SNN architecture trained with a spike representation achieves and accuracy of 94% and a precision of 0.92 at a 33 ms temporal window. Additionally, the proposed model demonstrates good performance compared to standard SNN benchmarks with lower parameter count while preserving higher computational efficiency. These results demonstrate the potential of SNNs in accurately distinguishing between fixations and saccades, capturing temporal structure and spike-based encoding of eye movement patterns. Moreover, this work highlights the potential of event-based vision for the development of a low-cost and low-latency biomarker for saccadic movement detection. Our approach also highlights the utility of Spiking Neural Networks in eye movement research in general and offers a valuable resource for future studies. The combination of event cameras and SNNs holds promise for advancing real-time and precise eye movement classification. With this study, we hope to provide a baseline for further research in this domain. The dataset and code for this work will be made available here upon review: https://github.com/Ikhadija-5/SNN_Classification (accessed on 30 January 2025).

## Figures and Tables

**Table 1:** Configuration and parameter count of the Spiking-ConvNet_SJ architecture.

Layer ID	SNN Layer	$c_{\mathrm{in}}$	$c_{\mathrm{out}}$	$k_{x}\times k_{y}$	$s_{x}\times s_{y}$	Parameters
1	Conv1	2	8	3×3	2×2	144
	Pool1	-	-	2×2	2×2	0
2	Conv2	8	8	3×3	1×1	576
	Pool2	-	-	2×2	2×2	0
3	Flatten	-	-	-	-	0
4	Dense1 + dropout (0.05)	11,264	103	-	-	1,160,592
5	Dense2 + dropout (0.05)	103	117	-	-	12,051
6	Output	117	2	-	-	234
**-**	**Total**	-	-	-	-	**1,173,597**

**Table 2:** Performance comparison between benchmark spiking models and our proposed Spiking-ConvNet at 33 ms, which is trained directly on spike trains against benchmarks utilizing a voxel cube representation with simulated timesteps (T=5) and micro timebins set to 2.

Model	Accuracy	Loss	Precision	Recall	F1-Score	Parameters (M)
SpikingDensenet	97.87	0.07887	0.9696	0.9884	0.9785	6.95
SpikingVGG11	98.06	0.1178	0.9697	0.9922	0.9808	9.22
SpikingVGG13	98.84	0.0623	0.9884	0.9884	0.9884	5.00
SpikingVGG16	99.03	0.0629	0.9847	0.9961	0.9904	14.72
SpikingSqueezenet	91.86	0.3231	0.8600	1.0000	0.9247	0.74
**Spiking-ConvNet (Ours)**	93.06	0.4034	0.9245	0.9225	0.9050	**1.17**

**Table 3:** Performance of Spiking-ConvNet across varying temporal resolutions on the EV-Eye dataset.

Temporal Resolution (ms)	FPS	Accuracy (%)	Loss	F1 Score
200	5.00	97.67	0.0089	0.9767
100	10.00	96.70	0.0165	0.9591
80	16.67	92.44	0.0324	0.9242
50	20.00	96.51	0.0179	0.8801
33	30.30	94.96	0.0221	0.9050
20	50.00	94.16	0.0292	0.9185

**Table 4:** Comparison of SNN and ANN metrics across layers at 33 ms temporal resolution.

	Shape	SNN (Spiking-ConvNe)		ANN	
		**Events**	**Synops**	**Activations**	**MACs**
Layer-0	(179, 129, 8)	524.11		184,728	
Layer-1	( 90, 65, 8)	367.31	524.11	46,800	184,728
Layer-2	( 88, 63, 8)	1232.50	26,446.17	44,352	3,369,600
Layer-3	( 44, 32, 8)	529.00	1232.50	11,264	44,352
Layer-4	( 1, 1, 103)	7.12	54,487.14	103	1,160,192
Layer-5	( 1, 1, 117)	2.00	833.33	117	12,051
Layer-6	( 1, 1, 2)	0.20	4.00	2	234
Total		2662.25	83,527.26	287,366	4,771,157

## Data Availability

The original data presented in the study were sourced from the publicly available EV-Eye repository (https://github.com/Ningreka/EV-Eye.git (accessed on 1 January 2025)) which provides event-based vision data for neuromorphic benchmarking. A subset of this dataset was manually annotated for our experiments and will be made publicly accessible via Figshare (https://figshare.com/articles/dataset/Ev-eye_Dataset_Annotated_into_Saccades_and_Fixations/30722108?file=59868794 (accessed on 30 January 2026 )) upon publication.

## References

[1] Lawand S.A. (2024). Eye tracking techniques and medical applications: A comprehensive review. Int. J. Sci. Res. Arch..

[2] Park S., Aksan E., Zhang X., Hilliges O. (2020). Towards end-to-end video-based eye-tracking. Proceedings of the European Conference on Computer Vision.

[3] Startsev M., Zemblys R. (2023). Evaluating eye movement event detection: A review of the state of the art. Behav. Res. Methods.

[4] Wong A. (2008). Eye Movement Disorders.

[5] Tahri Sqalli M., Aslonov B., Gafurov M., Mukhammadiev N., Sqalli Houssaini Y. (2023). Eye tracking technology in medical practice: A perspective on its diverse applications. Front. Med. Technol..

[6] Gilchrist I. (2011). Saccades. The Oxford Handbook of Eye Movements.

[7] Land M. (2012). Saccade. https://www.britannica.com/science/saccade.

[8] Laubrock J., Cajar A., Engbert R. (2013). Control of fixation duration during scene viewing by interaction of foveal and peripheral processing. J. Vis..

[9] Iddrisu K., Shariff W., Corcoran P., O’Connor N., Lemley J., Little S. (2024). Event Camera based Eye Motion Analysis: A survey. IEEE Access.

[10] Birawo B., Kasprowski P. (2022). Review and evaluation of eye movement event detection algorithms. Sensors.

[11] Aljaafreh A., Alaqtash M., Al-Oudat N., Abukhait J., Saleh M. (2020). A low-cost webcam-based eye tracker and saccade measurement system. Int. J. Circuits Syst. Signal Process..

[12] Eibenberger K., Eibenberger B., Roberts D.C., Haslwanter T., Carey J.P. (2016). A novel and inexpensive digital system for eye movement recordings using magnetic scleral search coils. Med. Biol. Eng. Comput..

[13] Aungsakul S., Phinyomark A., Phukpattaranont P., Limsakul C. (2012). Evaluating feature extraction methods of electrooculography (EOG) signal for human–computer interface. Procedia Eng..

[14] Reda R., Tantawi M., Shedeed H., Tolba M.F. (2019). Analyzing electrooculography (eog) for eye movement detection. Proceedings of the International Conference on Advanced Machine Learning Technologies and Applications.

[15] Hanke M., Mathôt S., Ort E., Peitek N., Stadler J., Wagner A. (2019). A practical guide to functional magnetic resonance imaging with simultaneous eye tracking for cognitive neuroimaging research. Spatial Learning and Attention Guidance.

[16] Niehorster D.C., Hessels R.S., Benjamins J.S. (2020). GlassesViewer: Open-source software for viewing and analyzing data from the Tobii Pro Glasses 2 eye tracker. Behav. Res. Methods.

[17] Onkhar V., Dodou D., De Winter J. (2024). Evaluating the Tobii Pro Glasses 2 and 3 in static and dynamic conditions. Behav. Res. Methods.

[18] Gallego G., Delbrück T., Orchard G., Bartolozzi C., Taba B., Censi A., Leutenegger S., Davison A.J., Conradt J., Daniilidis K. (2020). Event-based vision: A survey. IEEE Trans. Pattern Anal. Mach. Intell..

[19] Nunes J.D., Carvalho M., Carneiro D., Cardoso J.S. (2022). Spiking neural networks: A survey. IEEE Access.

[20] Yamazaki K., Vo-Ho V.K., Bulsara D., Le N. (2022). Spiking neural networks and their applications: A review. Brain Sci..

[21] Li X.S., Fan Z.Z., Ren Y.Y., Zheng X.L., Yang R. (2021). Classification of eye movement and its application in driving based on a refined pre-processing and machine learning algorithm. IEEE Access.

[22] Klein C., Ettinger U. (2019). Eye Movement Research.

[23] Bachurina V., Arsalidou M. (2022). Multiple levels of mental attentional demand modulate peak saccade velocity and blink rate. Heliyon.

[24] Vasiljevas M., Damaševičius R., Maskeliūnas R. (2023). A human-adaptive model for user performance and fatigue evaluation during gaze-tracking tasks. Electronics.

[25] Balcazar J., Orr J.M. (2024). EyeingUncertain Rewards: Pupil diameter tracks task-related arousal and error feedback in voluntary task-switching. Behavioural Brain Research.

[26] Rayner K. (1998). Eye movements in reading and information processing: 20 years of research. Psychol. Bull..

[27] Abi-Dargham A., Moeller S.J., Ali F., DeLorenzo C., Domschke K., Horga G., Jutla A., Kotov R., Paulus M.P., Rubio J.M. (2023). Candidate biomarkers in psychiatric disorders: State of the field. World Psychiatry.

[28] Sekar A., Panouillères M.T., Kaski D. (2024). Detecting Abnormal Eye Movements in Patients with Neurodegenerative Diseases–Current Insights. Eye Brain.

[29] Tsitsi P., Benfatto M.N., Seimyr G.Ö., Larsson O., Svenningsson P., Markaki I. (2021). Fixation duration and pupil size as diagnostic tools in Parkinson’s disease. J. Park. Dis..

[30] Leube A., Rifai K. (2017). Sampling rate influences saccade detection in mobile eye tracking of a reading task. J. Eye Mov. Res..

[31] Salvucci D.D., Goldberg J.H. Identifying fixations and saccades in eye-tracking protocols. Proceedings of the 2000 Symposium on Eye Tracking Research & Applications, Palm Beach Gardens.

[32] Zemblys R., Niehorster D.C., Komogortsev O., Holmqvist K. (2018). Using machine learning to detect events in eye-tracking data. Behav. Res. Methods.

[33] Fikri M.A., Santosa P.I., Wibirama S. A review on opportunities and challenges of machine learning and deep learning for eye movements classification. Proceedings of the 2021 IEEE International Biomedical Instrumentation and Technology Conference (IBITeC).

[34] Shurupova M.A., Aizenshtein A.D., Chistiakov S.N., Dolganov A.Y., Zhdanov A., Ivanova G.E. Applying the eye-tracking method for the classification of neurological disorders, mental diseases, and speech impairments based on machine learning: An overview. Proceedings of the 2023 IEEE Ural-Siberian Conference on Computational Technologies in Cognitive Science, Genomics and Biomedicine (CSGB).

[35] Mccarty C. (2022). Machine Learning for Event Detection in Eye-Tracking. https://osf.io/preprints/osf/29jye_v1.

[36] Wang C., Wang R., Leng Y., Iramina K., Yang Y., Ge S. (2024). An Eye Movement Classification Method based on Cascade Forest. IEEE J. Biomed. Health Inform..

[37] Lobão-Neto R., Brilhault A., Neuenschwander S., Rios R. (2022). Real-time identification of eye fixations and saccades using radial basis function networks and Markov chains. Pattern Recognit. Lett..

[38] Kastrati A., Plomecka M.B., Wattenhofer R., Langer N. Using deep learning to classify saccade direction from brain activity. Proceedings of the ACM Symposium on Eye Tracking Research and Applications.

[39] Wu C., Liaqat S., Cheung S.c., Chuah C.N., Ozonoff S. Predicting autism diagnosis using image with fixations and synthetic saccade patterns. Proceedings of the 2019 IEEE International Conference on Multimedia & Expo Workshops (ICMEW).

[40] Tobii Technology. Tobii Pro Glasses 3. 2025. https://www.tobii.com/products/eye-trackers/wearables/tobii-pro-glasses-3/.

[41] Chakravarthi B., Verma A.A., Daniilidis K., Fermuller C., Yang Y. (2024). Recent event camera innovations: A survey. Proceedings of the European Conference on Computer Vision.

[42] Li N., Chang M., Raychowdhury A. (2024). E-gaze: Gaze estimation with event camera. IEEE Trans. Pattern Anal. Mach. Intell..

[43] Ryan C., Elrasad A., Shariff W., Lemley J., Kielty P., Hurney P., Corcoran P. (2023). Real-time multi-task facial analytics with event cameras. IEEE Access.

[44] Iddrisu K., Shariff W., Little S. (2024). A framework for pupil tracking with event cameras. Proceedings of the IET Conference Proceedings CP887.

[45] Kang D., Lee Y.K., Jeong J. (2023). Exploring the potential of event camera imaging for advancing remote pupil-tracking techniques. Appl. Sci..

[46] Ryan C., O’Sullivan B., Elrasad A., Cahill A., Lemley J., Kielty P., Posch C., Perot E. (2021). Real-time face & eye tracking and blink detection using event cameras. Neural Netw..

[47] Iddrisu K., Shariff W., OConnor N.E., Lemley J., Little S. (2024). Evaluating Image-Based Face and Eye Tracking with Event Cameras. arXiv.

[48] Shariff W., Kielty P., Lemley J., Corcoran P. (2024). Spiking-DD: Neuromorphic event camera based driver distraction detection with spiking neural network. Proceedings of the IET Conference Proceedings CP887.

[49] Jiang Y., Wang W., Yu L., He C. (2024). Eye tracking based on event camera and spiking neural network. Electronics.

[50] Ren H., Zhou Y., Huang Y., Fu H., Lin X., Song J., Cheng B. (2023). Spikepoint: An efficient point-based spiking neural network for event cameras action recognition. arXiv.

[51] Barchid S., Allaert B., Aissaoui A., Mennesson J., Djeraba C.C. Spiking-FER: Spiking neural network for facial expression recognition with event cameras. Proceedings of the 20th International Conference on Content-based Multimedia Indexing.

[52] Lielamurs E., Ozols K. Spatio-temporal Object Detection with Deep Spiking CNNs Using Time-of-Flight Data. Proceedings of the 2024 19th Biennial Baltic Electronics Conference (BEC).

[53] Saquib T. (2022). Visual Tracking with Spiking Neural Networks in an Oculomotor Controller for a Biomimetic Model of the Eye.

[54] Kirkland P., Di Caterina G. Movement classification and segmentation using event-based sensing and spiking neural networks. Proceedings of the 2022 Sensor Signal Processing for Defence Conference (SSPD).

[55] Hasssan A., Meng J., Seo J.S. Spiking neural network with learnable threshold for event-based classification and object detection. Proceedings of the 2024 International Joint Conference on Neural Networks (IJCNN).

[56] Ahmed S.H., Finkbeiner J., Neftci E. Efficient Event-Based Object Detection: A Hybrid Neural Network with Spatial and Temporal Attention. Proceedings of the Computer Vision and Pattern Recognition Conference.

[57] Troconis L.G., Vella F., Freddi A., Monteriù A. SEEN: A Convolutional Spiking Neural Network for Efficient Pupil Coordinate Prediction from Event Data. Proceedings of the 2025 3rd Cognitive Models and Artificial Intelligence Conference (AICCONF).

[58] Yang Y., Xuan Z., Kang Y. TQ-TTFS: High-Accuracy and Energy-Efficient Spiking Neural Networks Using Temporal Quantization Time-to-First-Spike Neuron. Proceedings of the 2024 29th Asia and South Pacific Design Automation Conference (ASP-DAC).

[59] Teeter C., Iyer R., Menon V., Gouwens N., Feng D., Berg J., Szafer A., Cain N., Zeng H., Hawrylycz M. (2018). Generalized leaky integrate-and-fire models classify multiple neuron types. Nat. Commun..

[60] Bouanane M.S., Cherifi D., Chicca E., Khacef L. (2023). Impact of spiking neurons leakages and network recurrences on event-based spatio-temporal pattern recognition. Front. Neurosci..

[61] Team L.D. (2025). Dynamics, Neurons, and Spikes—Lava Documentation. https://lava-nc.org/lava-lib-dl/index.html.

[62] Shrestha S.B., Orchard G. (2018). Slayer: Spike layer error reassignment in time. Adv. Neural Inf. Process. Syst..

[63] Orchard G., Jayawant A., Cohen G.K., Thakor N. (2015). Converting static image datasets to spiking neuromorphic datasets using saccades. Front. Neurosci..

[64] Angelopoulos A.N., Martel J.N., Kohli A.P., Conradt J., Wetzstein G. (2020). Event based, near eye gaze tracking beyond 10,000 Hz. arXiv.

[65] Simpsi A., Aspesi A., Mentasti S., Merigo L., Ongarello T., Matteucci M. (2025). High-frequency near-eye ground truth for event-based eye tracking. arXiv.

[66] Blignaut P. (2009). Fixation identification: The optimum threshold for a dispersion algorithm. Atten. Percept. Psychophys..

[67] Cordone L., Miramond B., Thierion P. Object detection with spiking neural networks on automotive event data. Proceedings of the 2022 International Joint Conference on Neural Networks (IJCNN).

